# Environmental Enrichment Modulates Cortico-Cortical Interactions in the Mouse

**DOI:** 10.1371/journal.pone.0025285

**Published:** 2011-09-22

**Authors:** Angelo Di Garbo, Marco Mainardi, Santi Chillemi, Lamberto Maffei, Matteo Caleo

**Affiliations:** 1 Institute of Biophysics, CNR - National Research Council, Pisa, Italia; 2 Neuroscience Institute, CNR - National Research Council, Pisa, Italia; VU University, The Netherlands

## Abstract

Environmental enrichment (EE) is an experimental protocol based on a complex sensorimotor stimulation that dramatically affects brain development. While it is widely believed that the effects of EE result from the unique combination of different sensory and motor stimuli, it is not known whether and how cortico-cortical interactions are shaped by EE. Since the primary visual cortex (V1) is one of the best characterized targets of EE, we looked for direct cortico-cortical projections impinging on V1, and we identified a direct monosynaptic connection between motor cortex and V1 in the mouse brain. To measure the interactions between these areas under standard and EE rearing conditions, we used simultaneous recordings of local field potentials (LFPs) in awake, freely moving animals. LFP signals were analyzed by using different methods of linear and nonlinear analysis of time series (cross-correlation, mutual information, phase synchronization). We found that EE decreases the level of coupling between the electrical activities of the two cortical regions with respect to the control group. From a functional point of view, our results indicate, for the first time, that an enhanced sensorimotor experience impacts on the brain by affecting the functional crosstalk between different cortical areas.

## Introduction

The integration of sensory inputs is achieved through the interaction of different cortical areas [1], [2]. There is now considerable evidence that primary sensory cortices do not function as independent modules, but appear to functionally interact to provide integration between various modalities. For instance, the primary visual cortex (V1) is known to receive a functional input from primary auditory cortex, as demonstrated by the direct recording of auditory responses in visual cortical neurons [3]. Cross modal plasticity is particularly relevant in patients who are deprived of one sensory modality. For instance, early blind people display a higher tactile perceptual ability that depends on functional takeover of cortical visual areas by somatosensory inputs [4]. These data imply that brain circuits can undergo plastic rearrangements in response to changes induced by experience, such as those caused by pathological deprivation of one sensory modality. Functional integration is not restricted to cortices processing different sensory modalities, but appears to involve also motor areas. Particularly clear evidence of this integration comes from studies based on the experimental protocol of environmental enrichment (EE), which provides animals with an increased motor activity and sensory stimulation [5], [6]. The animals subjected to EE are reared in numerous social groups, in large cages where a variety of objects are present (toys, tunnels, platforms, running wheels, stairs, etc.). This results in a complex sensory stimulation and the opportunity for spatial and cognitive exploration, coupled to voluntary physical activity and social interaction, all factors that are absent in standard laboratory cages [7]. Many studies have shown that EE promotes molecular, anatomical and functional changes of neural circuits [7], [8], [9]. At the anatomical level, EE increases neurogenesis, soma size of neurons, dendritic arbor complexity and density of dendritic spines [7]. Functionally, EE results in an improvement of cognitive capabilities, accompanied by enhancement of markers of synaptic plasticity and transmission [7], [9], [10]. In sensory cortices, EE is able to dramatically affect the development of neuronal functional properties [9]; for example, the maturation of visual performance is accelerated in the visual cortex of rodents kept in EE since birth [11]. The combination of different sensory and motor stimuli is thought to be critical for the effects of EE; however, the precise way in which EE affects cortico-cortical interactions has not been investigated so far. In particular, motor activity is a very important component of EE [7]. Remarkably, EE drives the development of the visual system even if the animals are reared in complete darkness since birth [12].

These experimental results raise the possibility that inputs from other cortical areas, in particular motor regions, can regulate the development of the primary visual cortex (V1) in the absence of visual stimuli. Therefore, we first looked for an anatomical substrate for communication between motor and visual areas. We identified a monosynaptic projection linking secondary motor cortex (M2) and V1 in the mouse brain. Then, we analyzed simultaneous recordings of local field potentials (LFPs) from V1 and M2 of awake, freely-moving mice to quantify their synchronization level under standard and EE rearing conditions. To this aim, we used the cross correlation and the mutual information methods, that are typical tools for detecting coupling between complex signals [13], [14], [15], [16], [17], [18]. In addition, we also employed a new method to detect coupling between time series, called Slope Phase Coherence, that we introduce for the first time in this paper.

## Materials and Methods

### Ethics statement

All procedures were performed according to the guidelines of the Italian Ministry of Health for care and maintenance of laboratory animals (law 116/92), and in strict compliance with the European Communities Council Directive n. 86/609/EEC. Animal experimentation at the CNR Neuroscience Institute was approved by the Italian Ministry of Health (authorization # 129/2000−A). Specifically, the experiments described in this study were authorized by the Italian Ministry of Health via decree # 185/2009-B, released on November 4, 2009.

### Animal treatment

C57BL/6J mice were housed in an animal room with a 12 h/12 h light/dark cycle, with food and water available *ad libitum*. Pregnant dams were put either in standard or EE condition one week before delivery and pups were hatched at postnatal day (P) 25. The standard rearing condition consisted of a 26 X 18 X 18 cm cage housing 3 animals. The EE condition was achieved using a large cage (44 X 62 X 28 cm) containing several foodhoppers, one running wheel for voluntary physical exercise, and differently shaped objects (tunnels, shelters, stairs) that were repositioned twice a week and completely substituted with others once a week. Moreover, in the EE condition, two to three helper, non-pregnant females were added. For the EEG analyses, a total of 14 mice were used, 6 reared in environmental enrichment and 8 in the standard condition. Electrode implantation (see below) was performed at P60. Another five standard and three enriched animals were used for neuroanatomical tracings.

### Neuroanatomical tracing

To identify cortical areas monosynaptically connected with the primary visual cortex (V1), we used the neuronal tracer Cholera Toxin beta subunit (CTB, Sigma, USA). Mice were mounted on a custom-made stereotaxic apparatus, then a burr hole was drilled in the skull overlying V1. Stereotaxic coordinates corresponding to V1 were 0.0 mm anteroposterior and 2.5 mm mediolateral to the lambda point. To maximize the spatial specificity of the injection, a minute amount (50 nl) of CTB solution (1% in water) was injected at a depth of 600 µm. Injection was performed by using a 0.5 µl Hamilton syringe (Hamilton, USA) filled with mineral oil and plugged to a glass injection pipette. After allowing 3 days for transport of CTB to neuronal somata and processes, animals were transcardially perfused with 50 ml of 4% PFA, then brains were frozen and cut using a cryostat (Leica, Germany) to obtain 50 µm-thick coronal sections. CTB labeling was visualized by means of immunohistochemistry. Free-floating sections were blocked in 5% normal rabbit serum (NRS), 2.5% bovine serum albumin (BSA), 0.3% Triton X-100 in PBS for 2 hrs at RT. Incubation with primary antibody was performed with 1∶4000 anti-CTB made in goat (Calbiochem, USA), 2% NRS, 2.5% BSA, 0.1% Triton X-100 in PBS, overnight at 4°C. Subsequently, sections were transferred in a solution containing 2% NRS, 2.5% BSA, 1% Triton X-100 and 1∶500 anti-goat biotinilated secondary antibody in PBS, for 2 hrs at RT. This was followed by incubation for 1 h in ABC kit (Vector Labs) and final detection with DAB reaction kit (Vector Labs). Sections were finally mounted on glass slides, dehydrated and sealed with DPX mounting medium (VWR International, UK). Images were acquired using a CCD camera (Zeiss, Germany) mounted on an Axioskop microscope (Zeiss).

Countings of retrogradely labeled cells in M2 were performed using a CCD camera (MBF Bioscience, Germany) mounted on a Zeiss Axioskop (Zeiss, Germany) microscope and the StereoInvestigator software (MBF Bioscience). For each coronal section comprising M2, the area containing stained cells was outlined and its area measured. Then, the number of CTB-positive cells was counted and their density calculated (cells/mm^2^). The density value for each experimental case was obtained by averaging the data from at least 6 sections.

### Local field potential recordings in freely moving mice

**Figure 1:**
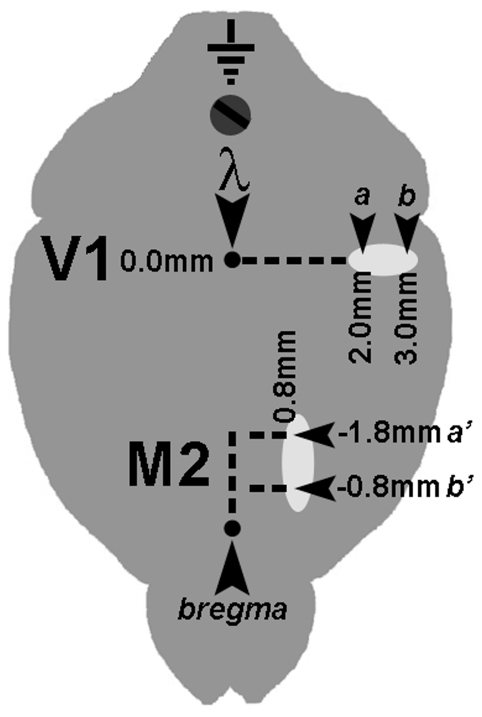
Schematic diagram showing the disposition of recording sites. Two electrodes were placed in either the secondary motor (M2) and primary visual (V1) cortical areas with a 1 mm spacing to achieve the necessary specificity for sampling local field potentials; a ground reference screw was placed in the occipital bone, over the cerebellum. Local field potentials were acquired as the differential between electrodes a and b for V1, a' and b' for M2, respectively.

**Figure 2:**
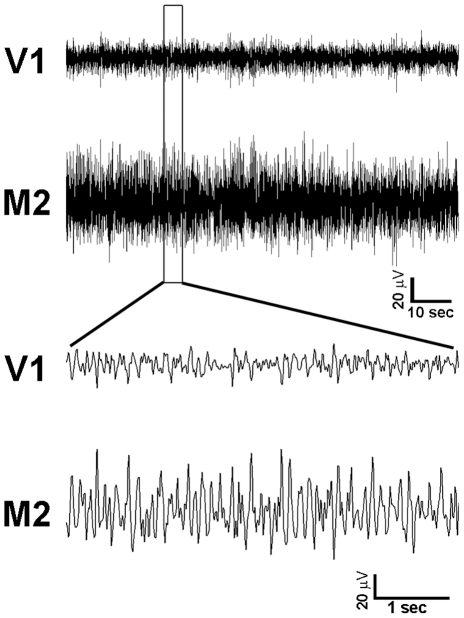
Representative traces of LFP signals recorded in primary visual (V1) and secondary motor (M2) cortices. The upper panel shows the typical aspect of the recorded LFP signals over a 100 sec-time epoch; the inset indicates a 5 sec period that is magnified in the lower panel.

Local field potential (LFP) recordings were performed in awake, freely moving mice using an adaptation of the protocol described by Antonucci et al. [19]. Low-impedance recording electrodes made of nichrome wire (120 µm thick) were tin-soldered to a multipin socket to create an array comprising four electrodes; the fifth position of the socket received an insulated copper ground cable. Under avertin anaesthesia (0.01 ml/g) and after placement in a stereotaxic apparatus, the skull was exposed and four burr holes were drilled in the skull at given positions (see below), paying attention not to damage the underlying dural surface. The multipin socket was held by an adjustable manipulator and the electrodes were put in place, establishing an electrical contact without lesioning the dura mater. LFPs were sampled by placing the tips of a couple of electrodes in the same cortical area, spaced by 1.0 mm to achieve detection of local electrical activity confined between the two sites. A ground screw was positioned on the occipital bone and connected with the ground cable, while an additional screw was installed on the frontal bone to provide further strength to the implant. The whole device was secured in place by means of dentistry acrylic cement (Paladur, Pala, Germany). Stereotaxic coordinates were (i) between 2.0 mm and 3.0 mm lateral (L) and 0.0 mm anteroposterior (AP) to lambda for V1; (ii) 0.8 mm L and between −0.8 mm and −1.8 mm AP to bregma for secondary motor cortex (M2) [20]. A representation of the positioning of the recording electrodes is given schematically in Figure 1. Animals were returned to their home cage and recordings were done after allowing 3 to 5 days for recovery from surgery; the animal was habituated for 1 hr to the test cage, then a 1 hr recording session was performed, using a digital acquisition system. No differences in behavior were detected during LFP recordings between EE and control mice (see below). The hardware was composed of a custom-made buffer to eliminate movement artifact from the signal, an amplifier and an acquisition card (National Instruments, USA), plugged via USB to a personal computer. The custom-made acquisition software was based on the LabView platform (National Instruments). Cortical LFP signals were acquired with a sampling rate of 100 Hz as the differential between the two adjacent electrode sites placed in the same cortical area, 50000X amplified and 0.3–30 Hz band-passed. Representative examples of the recorded LFPs are shown in Fig. 2.

### Behavioural analysis

Two additional experimental groups (SC, n = 7; EE, n = 4) were used to quantify the exploratory behaviour of EE and SC mice, by using the EthoVision XT software (Noldus, Leesburg, USA) and a CCD camera (Panasonic, Japan). Animals were placed in the same cage that was used for LFP recordings (see above) and allowed for a habituation period of 1 hour, then their movements filmed for the same duration of an electrophysiology recording session (1 hour). The acquired tracks were used to quantify representative parameters of the exploratory activity of EE and SC animals when placed in the LFP recording cage.

### Data description

Each data set consists of a bivariate time series representing the LFPs simultaneously recorded from visual (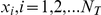) and motor cortex (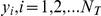).The value of *N_T_* is 300000 points. As an example in Figure 2 are reported some traces of the recorded signals. Before the analysis, all time series were visually inspected to confirm the absence of recording artifacts. Moreover, each time series was normalized to zero mean and unit standard deviation. Two group of data were used for the analysis: the data of the control group (SC) obtained from 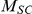 = 8 mice and the data of the EE group recorded from 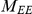 = 6 animals. To satisfy the request of stationarity all time series were partitioned in half-overlapping windows each containing 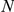 = 5000 data. If 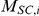 is the number of half-overlapping windows of 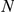 points contained in the 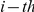-bivariate time series of the control group, then their total number is 
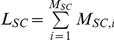
. Similarly, 
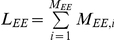
 for the EE group. To quantify the synchronization level of LFP signals from V1 and M2 cortex several methods of bivariate time series analysis were employed [13], [15].

### Mean Cross Correlation

Let be 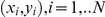 two discrete signals, then the cross correlation function at time lag 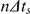 is defined as 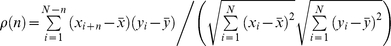, where 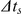 is the signal sampling interval and 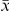 (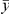) is the mean value of the signal 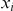 (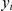). To quantify the interdependence properties between the LFP recordings in V1 and M2 the mean value of the cross correlation over time lags was employed. In particular, the definition of this measure adopted is: 
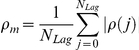
, where 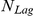 is the number of time lags. The quantity 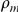 quantifies the level of the linear correlation between two signals over a given time window. Here it assumed that 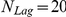 and the corresponding time window is 190 msec (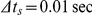). This choice for the value of 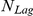 is an acceptable compromise between computational advantage and physiological relevance. It is worth noting that a similar measure was used to quantify the degree of interdependence between electroencephalogram recordings in [17]. Let 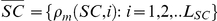 be the set of all values of 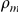 obtained for the data of the control group (SC), and 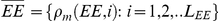 the corresponding set for the EE group. Then the mean and standard error of the set of values 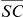 and 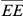 were estimated. To determine whether the results are consistent among animals, we also computed the mean 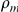 for each individual animal and the averages per experimental group were statistically compared. The same protocol was adopted to present the results of the other synchronization measures (Mutual Information and Slope Phase Coherence, see below).

### Mutual Information

The value of the mean cross correlation measures the strength of the coupling between two signals arising from linear correlations. To quantify both linear and nonlinear correlations between the LFPs of the V1 and M2 cortex, the mutual information (MI) analysis was used [21]. Let be 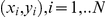 a bivariate time series. The MI between these signals is estimated by partitioning the signal ranges in bins of equal size, and by applying the following formula: 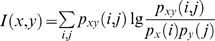 where 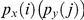 is the probability to find the value of the random variable 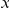 (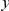) in the *i* - th (*j* - th) bin and 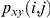 is the corresponding joint probability [21], [22]. To increase the reliability of the above approach another independent method of estimating MI, based on the *k*-neighbor statistics, was employed [22]. In this case the estimate of the mutual information between the two signals is given by: 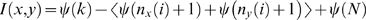, where 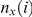 (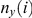) is the number of points 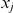 (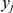) whose distance from 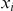 (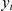) is strictly less than 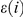, the symbol 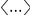 denotes the average value, 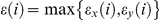 and 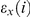 (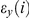) is the distance of 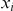 (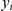) from its k-nearest neighbor, 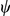 is the digamma function [22] and satisfies 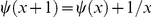 and 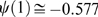. Here, both methods were used and compared to detect nonlinear correlations between the data from the V1 and M2 cortex. To show the results, the mean value and standard error of MI are computed by using the same approach as in section Mean Cross Correlation (see above).

### Slope Phase Coherence

The LFP signal is believed to represent the collective electrical activity of many neurons [23]. Therefore, it is expected that the rate of change of this recording contains information on the amount of synaptic intercommunication between neurons. Thus, to quantify the coupling level between the signals from V1 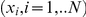 and M2 cortices 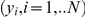 we propose here a very simple method. Let be 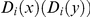 the approximation of the derivative of the discrete signal 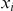 (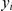) in the *i*-th point. Here we use quadratic polynomial interpolation to estimate the derivatives, and for each signal the following definition of the phase in the *i*-th point is used: 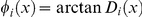 and 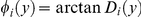. Because the method employs the amplitudes of the time series, it is extremely important that both recordings are normalized. A suitable choice, that we adopted here, is to normalize both signals to zero mean and unit standard deviation. Then the degree of interdependence between the two time series is quantified by the values of the mean Slope Phase Coherence (SPC) defined as 
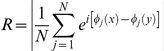
. For uncorrelated signals the value of the above quantity is close to zero, while it approaches 1 when 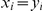, (*i* = 1,..,*N*). To show the results, the mean value and standard error of SPC are computed by using the same approach as in section Mean Cross Correlation (see above).

## Results

### Monosynaptic connections between motor and visual areas in the mouse

**Figure 3:**
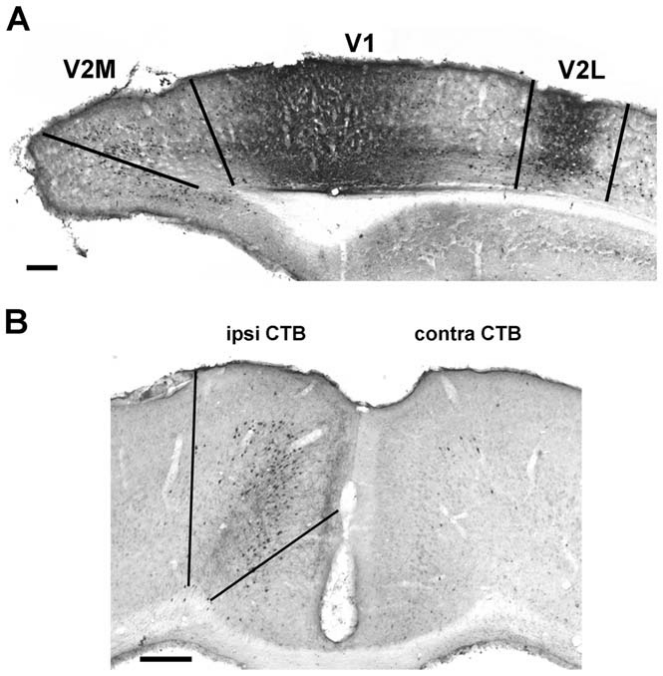
Identification of monosynaptic connections between the secondary motor cortex and the primary visual cortex. A) Image showing the confinement of the Cholera Toxin beta-subunit (CTB) injection site to the primary visual cortex (V1), whose limits are marked by black lines. The labeled spot in the lateral part of secondary visual cortex (V2L) reflects anterograde and retrograde transport via intracortical connections. Dorsal is up and lateral is to the right. This histological section corresponds to the coronal plane at −3.80 mm A/P with respect to bregma in the atlas by Paxinos and Franklin [20]. B) Representative coronal section showing retrogradely labeled cells in secondary motor cortex (M2) ipsilateral (left) and contralateral (right) to the CTB-injected V1. Borders of the ipsilateral M2 are marked by black lines. This histological section corresponds to the coronal plane at 0.02 mm A/P with respect to bregma in the atlas by Paxinos and Franklin [20]. Scale bars are 200 µm.

To identify a neuroanatomical substrate for communication between motor and visual areas, we stereotaxically injected a minute amount (50 nl) of the neuronal tracer cholera toxin beta subunit (CTB) into the primary visual cortex of adult (P60) mice subjected to either EE or standard rearing from birth. We verified that the tracer remained confined between the anatomical boundaries of V1 (Fig. 3A). Examination of anterior brain sections containing motor areas revealed a thin stripe of retrogradely labelled neurons in the medial part of the frontal cortex (Fig. 3B). We identified this area as the secondary motor cortex (M2) in the atlas by Paxinos and Franklin [20]. Labelling was particularly concentrated in the hemisphere ipsilateral to the injection, but some cells were also stained in the contralateral M2 (Fig. 3B). A network of CTB-positive fibers was also evident in the ipsilateral M2, suggesting anterograde transport of the tracer from V1. Cell countings in the ipsilateral M2 revealed no significant difference in the density of retrogradely labeled neurons between SC and EE mice (SC n = 5, 551±86 cells/mm^2^ and EE n = 3, 449±38 cells/mm^2^, respectively; Student's *t* test P = 0.420). We also screened the entire cortical mantle for other areas showing CTB-stained cell bodies; a comprehensive list, together with a qualitative assessment of the labeling density can be found in the Table S1.

On the basis of this neuroanatomical evidence, we went on to investigate whether functional interactions between V1 and M2 are regulated by an enriched sensorimotor experience. To this aim, we implanted bipolar electrodes in M2 and V1 to simultaneously record local field potentials (LFPs) from these two regions in freely moving, adult mice subjected to either EE or standard rearing.

**Figure 4:**
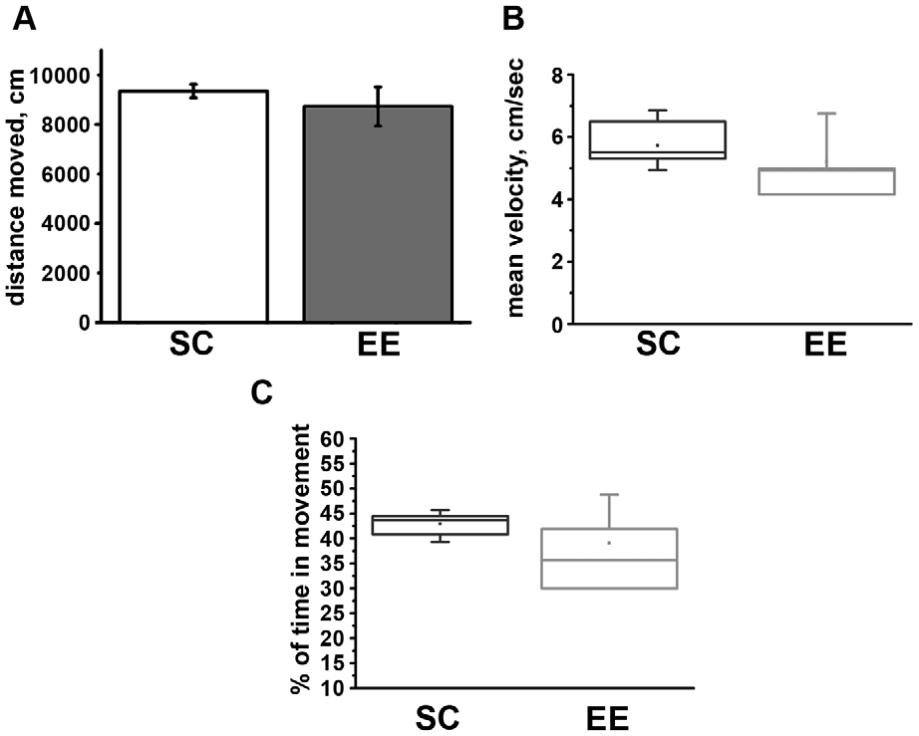
Exploratory activity of EE and SC animals. When placed in the LFP recording cage, EE and SC animals did not display any significant behavioural difference with regard to distance moved (A, Student's *t* test, P = 0.384), mean velocity of movement (B, Mann-Whitney Rank Sum test, P = 0.230) and percentage of time spent moving (C, Mann-Whitney Rank Sum test, P = 0.412). The horizontal lines in the box chart denote the 25th, 50th, and 75th percentile values. The error bars denote the 5th and 95th percentile values, while the square indicates the mean of the data.

To ascertain that the behaviours of SC and EE were similar when placed in the recording cage, an independent subset of animals were monitored with a camera and subjected to quantitative evalutation of their exploratory activity (see Methods section). Neither the total distance moved nor the mean velocity of movement were significantly different between the two groups (Fig. 4A, B; Student's *t* test, P = 0.384 and Mann-Whitney Rank Sum test, P = 0.230, respectively); importantly, also the percentage of time spent moving was similar in SC and EE mice (Fig. 4C; Mann-Whitney Rank Sum test, P = 0.412). This suggests that our measures of functional coupling between M2 and V1 (see below) reflect the nature of synaptic connectivity between these areas and are not biased by differences in behaviour during the recording period.

The LFPs were analyzed by using different methods of linear and nonlinear analysis of bivariate time series [13], [15], [18]. The results from these analyses (maximum cross correlation, mutual information, and slope phase coherence) are described in turn below.

### Power Spectrum

**Figure 5:**
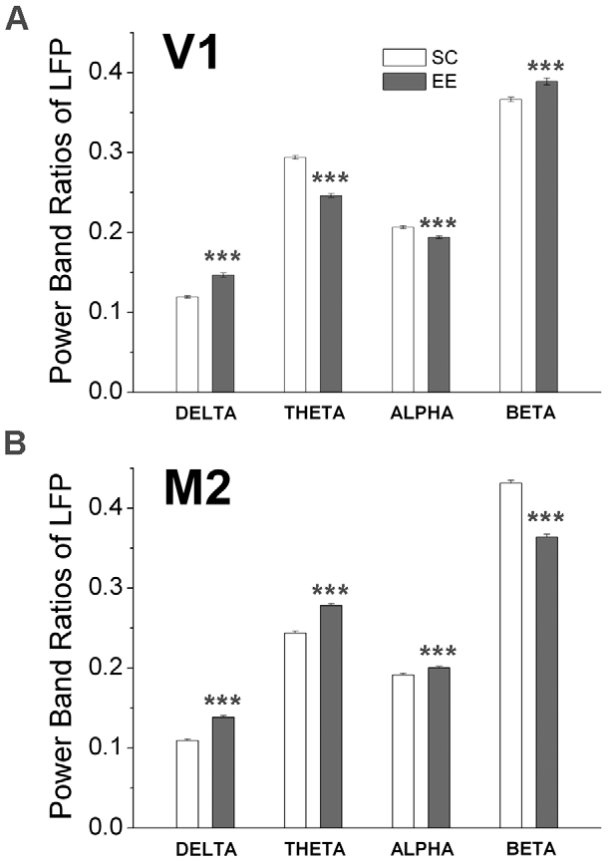
Power band ratios of LFP signal. A) Mean values and standard errors of the power band ratios corresponding to V1 in EE and control mice. B) Mean values and standard errors of the power band ratios corresponding to M2 in EE and control mice.

As preliminary step the spectral properties of the LFP signals recorded in V1 and M2 were investigated. In particular, the power band ratios of the LFP recordings were estimated for the control (SC) and EE condition. These quantities are defined as follows: 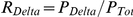, 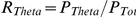, 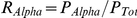 and 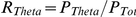 where 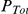 is the total power, while 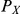 (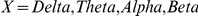) is the power in the corresponding band (delta, 0–4 Hz; theta, 4–8 Hz; alpha, 8–13 Hz; beta, 13–30 Hz). Each time series was divided in half-overlapping windows of 4096 data points and the Fourier spectrum averaged over all partitions. Then the mean value and the standard error were computed. In Figure 5 are reported the corresponding results for V1 and M2 cortices. A consistent finding was an increase in the power of the delta band in both V1 and M2 of EE mice as compared to controls. The application of the t-test shows that the differences between these power ratios are statistically significant (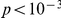).

### Mean cross correlation

**Figure 6:**
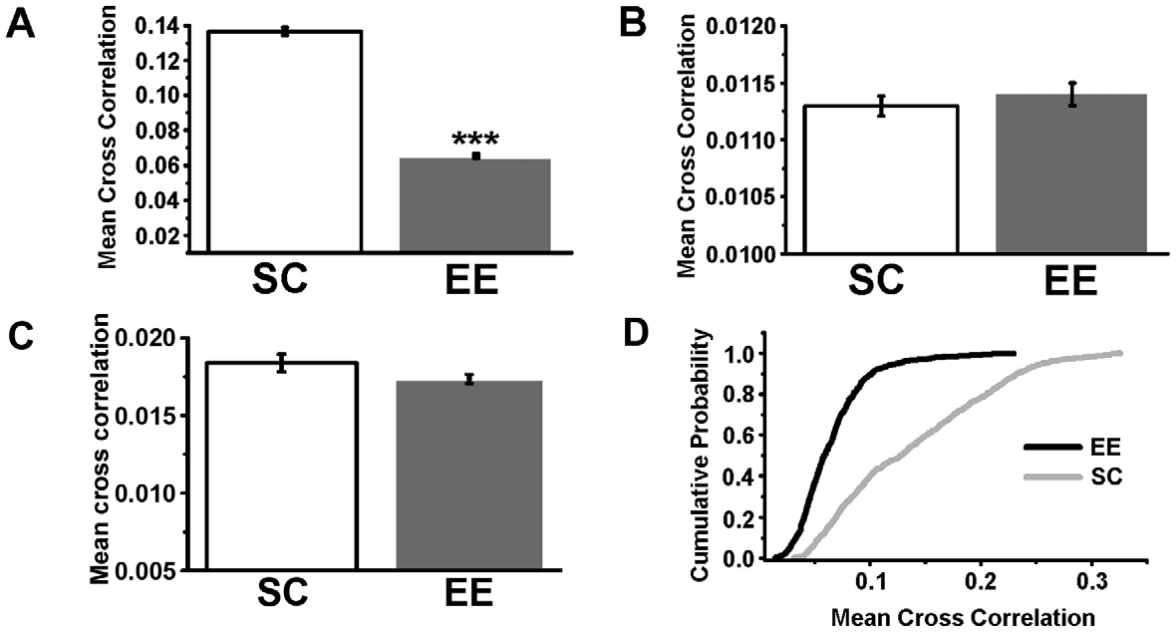
Mean linear cross correlation values between local field potentials from motor and visual cortices. A) Mean values of 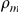 for the EE and control data. B) Same as A), but after random shuffling of the data. C) Same as A), but after random shuffling of data epochs. D) Cumulative probability distribution for the EE (black tick line) and control (gray line) data.

To quantitatively assess the level of interaction between local neural activities in M2 and V1, we began by using a linear measure, the mean cross correlation. In panel A) of Figure 6 are reported the average values of the mean cross correlation (± standard error) for enriched (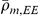) and control animals (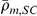). The difference between the average values of the mean cross correlation for the two groups of data is statistically significant: the application of the t-student and the non-parametric Mann-Whitney tests gives *p*<10^−^^3^. In particular 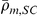 is greater than 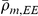 and this implies that the EE condition promotes the reduction of the synchronization level between V1 and M2 cortices. To assess if the observed synchronization levels came from chance, randomly shuffled surrogate data were used and the corresponding results are plotted in panel B) of Figure 6. The surrogate data corresponding to the LFP recording in V1 (M2) were obtained by independent random shuffling the whole time series. The statistical comparison of the results of panels A) and B) indicate that the inequality 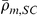
>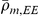 cannot arise from chance. Random shuffling of the data destroys all correlations in the signal and thus the corresponding power spectrum is whitened. Therefore, to preserve the power spectrum of the original signal and to make a more meaningful comparison between the values of the mean cross correlation for the EE and SC conditions, we used different surrogate data. In particular, the values of the mean cross correlation were estimated on the data obtained by shuffling all epochs of the signals, i.e. by selecting randomly the time windows of the LFP recording in V1 and M2. The corresponding results are reported in panel C of figure 6 and are consistent with those obtained with the random shuffling method (Fig. 6B). With both shuffling methods the differences in the crosscorrelation level between SC and EE animals are erased; moreover, the correlation indices of shuffled data are dramatically decreased with respect to the original LFP time series. The cumulative probability distributions of the two sets 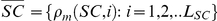 and 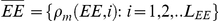 were also estimated and the corresponding results are reported in panel D) of Figure 6. The values of 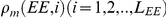 fall within the interval (0.06, 0.21), while those of 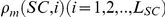 are distributed in a larger region. The two distributions were statistically compared by using the non-parametric Kolmogorov-Smirnov test [24] and the corresponding result is that they are different (*p*<10^−3^). In conclusion the above findings suggest that the EE condition promotes the segregation of local activity of the two cortices leading to the decrease of their synchronization level.

**Figure 7:**
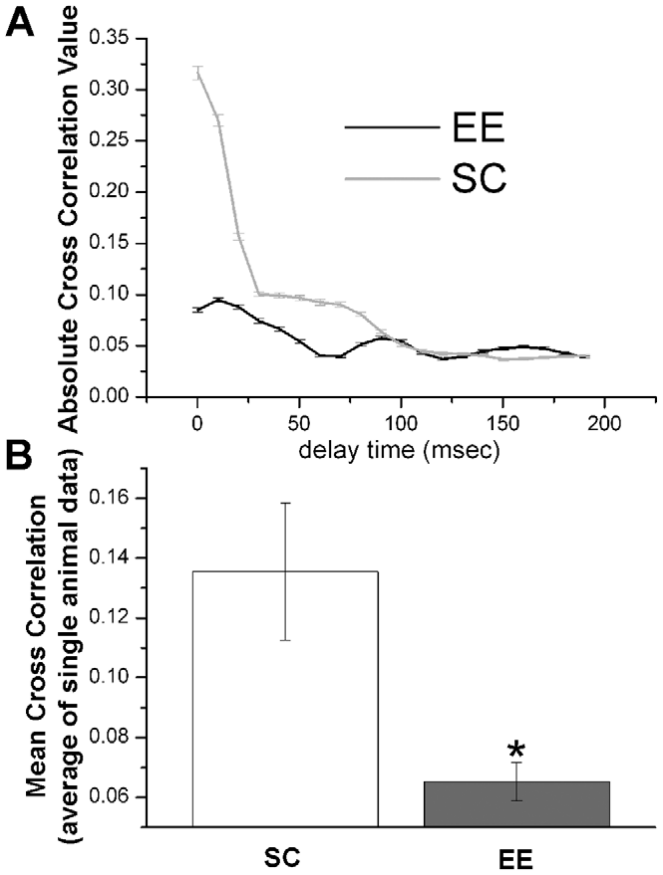
Absolute value of the linear cross correlation against the time lag and comparison of the population average. A) Dependence of the absolute value of the cross correlation function on the time lag. B) Mean cross correlation obtained by averaging the values of this coupling measure estimated for each mouse of the EE and SC groups.

To get more information on linear correlations properties between V1 and M2 signals, in the panel A of Figure 7 are plotted the values of 
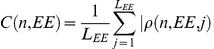
 and 
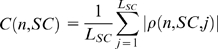
 as a function of the time lag. The quantity 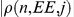 (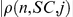) is the absolute value of the cross correlation at time lag 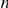 in the j-th windows for EE (SC) condition; the errorbars represent the corresponding standard errors. These data show that for time lags below 100 msec the values of the cross correlation for the SC is higher than for EE condition. Moreover 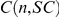 does not exhibit an oscillatory time course as does 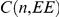. Lastly, the values of 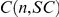 are distributed on a larger interval of those corresponding to the EE condition. In summary these results indicate that the EE rearing conditions affects the correlation properties between the LFP recordings in V1 and M2 in the time scale of tens of msec, which likely corresponds to the time required for the monosynaptic communication between the two areas.

An important point is how the individual mice compare with respect to the above measure. In other words, let be 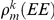 (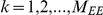) the value of the mean cross correlation for the *k*-th mouse of the EE group, and 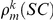
(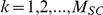) the corresponding quantity for the *k*-th mouse of the control group. Then, the mean values of the two groups of data were computed and the corresponding results are reported in the panel B of figure 6. The application of both the t-test and the Mann-Whitney indicates that the mean of the 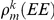 values is lower than that of the control group (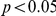). Thus, a consistent decrease in the mean cross correlation is observed in enriched mice as compared to controls.

### Mutual information

**Figure 8:**
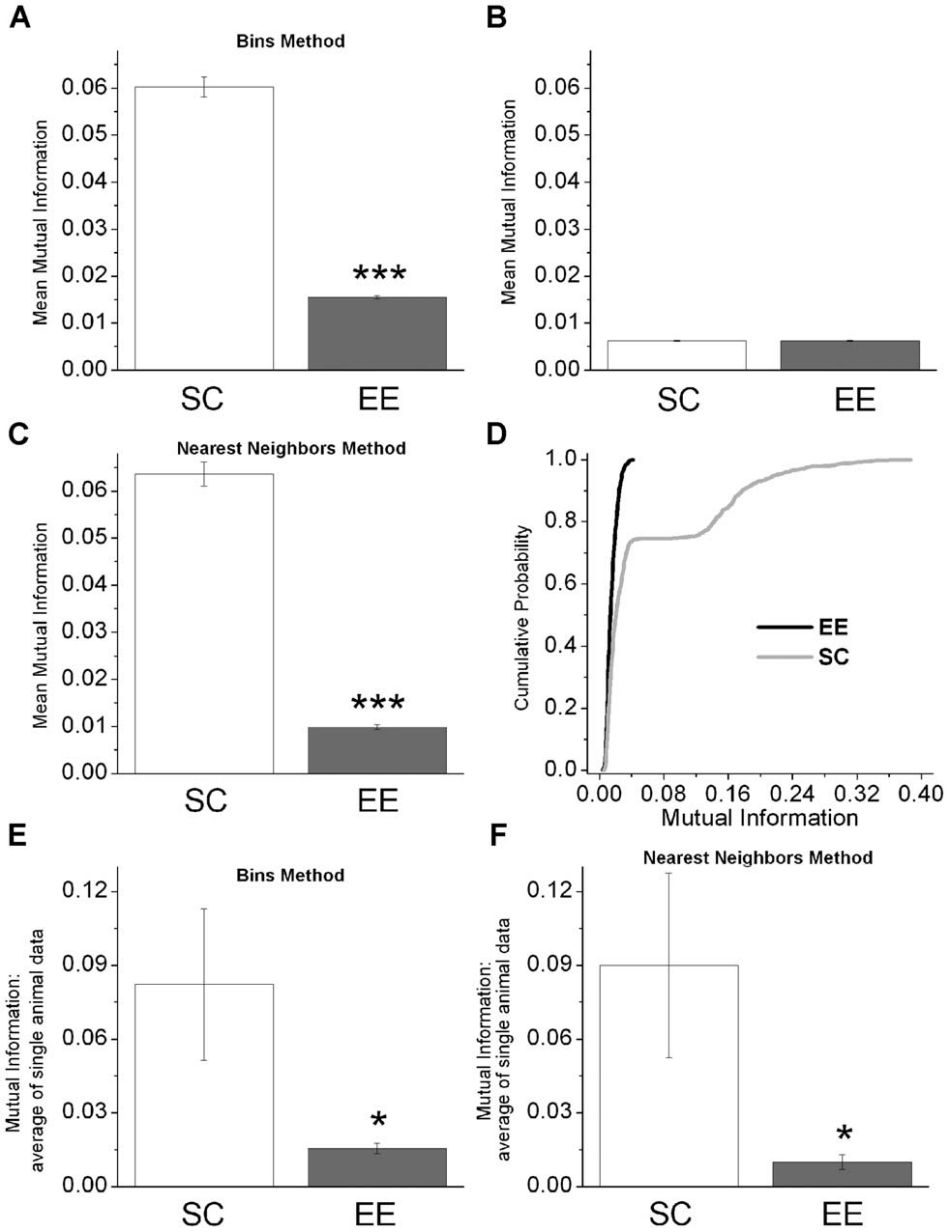
Mutual information values between local field potentials from motor and visual cortices. A) Mean values of 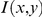 for the EE and control data obtained by data binning (the number of bins is equal to 10). B) Same as A), but after random shuffling of the data. C) Mean values of 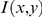 for the EE and control data obtained by using the nearest neighbor method to estimate the mutual information value (the number of neighbors is equal to 3). D) Cumulative probability distribution of the mean values of 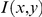 (using the binning method) for the EE (black tick line) and control (gray line) data. E) Mean value of the mutual information (using the bins method) obtained by averaging the values of this coupling measure estimated for each mouse of both EE and SC groups. F) Mean value of the mutual information (using the nearest neighbors method) obtained by averaging the values of this coupling measure estimated for each mouse of the EE and SC groups.

Next, the mutual information (MI) was used as a further measure of coupling between two signals (see Materials and Methods section). The mutual information is a quantity that measures the mutual dependence of two variables, taking into account both linear and nonlinear correlations. First the binning method was used to estimate the MI and the results are plotted in panel A) of Figure 8. The application of both the t-test and the non-parametric Mann-Whitney test shows that the difference between the control and the EE groups is statistically significant (*p*<10^−3^). In particular, the EE condition showed lower values of MI as compared to controls. As in the previous case the data were randomly shuffled and the corresponding MI values are reported in panel B) of Figure 8. The statistical comparison of these MI values indicates that the inequality 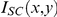
>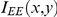 cannot arise from chance. The results obtained with surrogate data in which the epochs of the signals are randomly selected are in keeping with the above conclusion (data not shown). Then, the MI values were estimated by using the nearest neighbor approach and the corresponding results, reported in panel C), are in agreement with those of panel A). The cumulative probability distributions of the MI values are reported in panel D) of Figure 8. The application of the non-parametric Kolmogorov-Smirnov test [24] indicates a statistically significant difference (p<10^−3^).

As in the case of the mean cross correlation, the mutual information was estimated for each mouse and the corresponding values were averaged. The results obtained for the EE and control group are reported in panels E and F of Figure 8. In panel E are plotted the mean values and standard errors obtained with the bins method, while in panel F those obtained with the nearest neighbors method. The results are consistent among them and with those reported in panel A and C; the application of the t-test and the Mann-Whitney test indicates that the mean values of the mutual information are statistically different (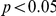).

### Slope phase coherence

**Figure 9:**
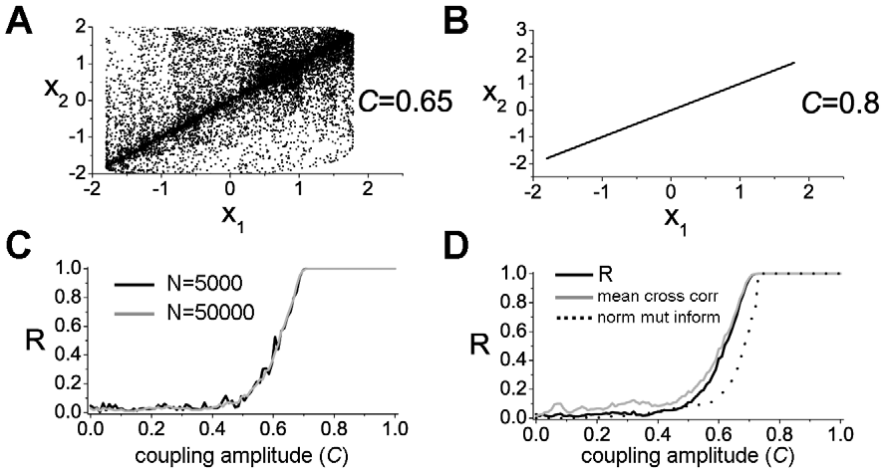
Test of the slope phase coherence method by using a pair of coupled Henon maps. A) 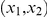 projection of the whole attractor for coupled maps (*C* = 0.65). B) Same as A), but with *C* = 0.8. C) Values of the slope phase coherence (*R*) against the coupling amplitude; the black line corresponds to *N* = 5000 data points, while the gray line to *N* = 50000. D) Comparison of the mathematical behaviour of the three synchronization measures. Slope phase coherence (black line), maximum cross correlation (gray line) and normalized mutual information 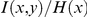 (dotted line) against the coupling amplitude; the mutual information was computed by using the binning method (the number of bins is equal to 10). For the data shown in panel D) the number of points of each bivariate time series was *N* = 50000; 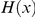 is the Shannon entropy of the signal 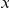.

We then employed a novel method of analysis, the Slope Phase Coherence (SPC, see Materials and Methods section), to quantify the degree of coupling between the simultaneous LFPs recorded in V1 and M2. SPC is based on the rate of change of the LFP recording as a measure of the amount of synaptic intercommunication between two neuronal populations. As a preliminary control to test the method, we studied bivariate data 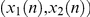 obtained from a pair of coupled Henon maps: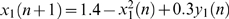, 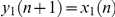 and 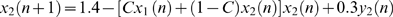, 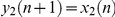. The intensity of the coupling is *C* and, according to previous literature [25], [26], varies between 0 and 1. The impact of the coupling strength on the level of correlation between 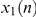 and 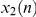 is shown in panels A) and B) of Figure 9, while in panel C) the values of *R* are reported against *C*. It is worth noting that the performance of the SPC method to detect the coupling between signals in not strongly sensitive to the size of the data sets (see panel C of Figure 9). Lastly, in panel D) of Figure 9, the capabilities of the three synchronization measures (mean cross correlation, mutual information and slope phase coherence) to detect the coupling between 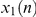 and 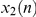 are compared. These three independent measures appear to give overlapping results (Fig. 9D). Altogether, the previous results indicate that the SPC method is suited to detect the coupling between two time series.

**Figure 10:**
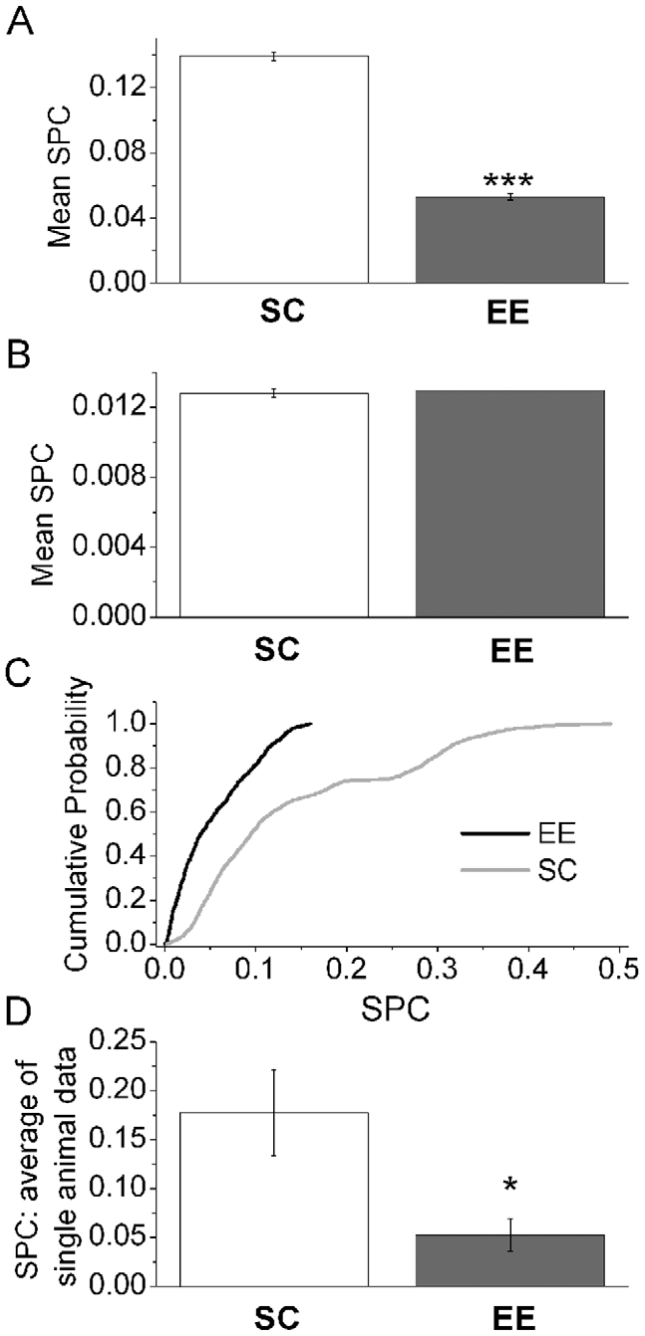
Slope phase coherence (SPC) values between local field potentials from motor and visual cortices. A) Mean values of SPC for the EE and control data. B) Same as A), but after random shuffling of the data. C) Cumulative probability distribution of the mean values of SPC for the EE (black tick line) and control (gray line) data. D) Mean values of the SPC quantity obtained by averaging the values of this coupling measure estimated for each mouse of the EE and SC groups.

This method was used to analyze the LFP signals from V1 and M2 and the corresponding results are reported in Figure 10. The results of panel A) show that the level of synchronization between signals in the EE condition is smaller than in standard-reared animals (*p*<10^−3^), in agreement with the previous cross correlation and mutual information analyses. The results obtained with the SPC method by using randomly shuffled data are reported in panel B) of Figure 10. Similar results were obtained with surrogate data in which the epochs of the signals were randomly selected (data not shown). In panel C) of Figure 10 the cumulative probability distributions of the R values, for both EE and control, are plotted. The R values for the EE group are smaller than 0.15, while for the control group they are distributed across a larger interval. The two distributions were statistically compared by using the non-parametric Kolmogorov-Smirnov test [24] and the result shows that they are significantly different (p<10^−3^). As for the previous cases, the SPC value was estimated for each mouse and then the corresponding values were averaged. The results obtained for the EE and control group are reported in panel D of Figure 10. The results are in keeping with those reported in panel A and the application of the t-test and the Mann-Whitney test indicates that the corresponding mean value for the EE group is lower than that for the control group (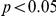).

## Discussion

EE is an experimental paradigm that is widely used to provide animals with a complex sensorimotor stimulation consisting of physical activity, different learning experiences and social interaction. Several experiments have shown that exposure to EE has potent ef­fects on neural circuitry in both the developing and adult brain [7]. Major effects of EE have been found in the hippocampus and sensory cortices [27], [28], but subcortical structures are affected as well [10]. It is widely held that the effects of EE result from the unique combination of the various stimulating factors (motor, sensory, social and cognitive) that are included in this protocol. However, no previous studies have examined how the interaction between different cortical areas is sculpted by an enriched experience during development. In particular, we focused our attention on the interaction between visual and motor areas, as it has been demonstrated that the effects of EE on the development of V1 are observed even if the animals are kept in complete darkness [12]. These data indicate that inputs from other cortical regions, such as motor regions, can represent the substrate of the actions of EE on functional maturation of the visual cortex. In this study, we analyzed LFP recordings in V1 and M2 cortices to understand how the EE condition impacts their functional interaction. The choice of analyzing V1 and M2 cortices was prompted by our anatomical observations, indicating a direct connection between these areas. The motor area containing retrogradely labelled cells after V1 injection (named M2 in the atlas by Paxinos and Franklin [20]) has been identified also in rats (see Fig. 2A in [29]). Studies in rats suggest that this area may be involved in the control of orienting and exploring behaviors, in addition to the control of eye movements [29], and may therefore serve as a hub for integrating visuo-motor activities. Thus, it was of interest to examine whether EE affects functional coupling between V1 and M2. To carry out this analysis, we used independent methods to quantify the degree of linear and nonlinear correlation between the LFPs recorded in the two regions, which was used as a measure of synchronization. It is important to point out that with the word synchronization, we refer to those dynamical states where two or more dynamical systems (in this case, V1 and M2) adjust their properties to share some common behaviour [14], [18]. We chose to analyze LFPs, as they represent the collective synaptic activity of neurons in the sampled area. We did not attempt to quantify correlations in firing of individual neurons, as this was thought to be less informative, considering the great number of functionally distinct units in the cortex and their variable cortico-cortical connectivity. The results of our analysis indicate that the EE rearing condition promotes a decrease of the synchronization level between V1 and M2 cortices. In particular, we noted a reduction in the cross-correlation that was maximal over the time window of a few tens of ms, consistent with the time required for monosynaptic communication between V1 and M2. Moreover, the different shapes of the cumulative probability distributions (Figs. 6D, 8D and 10C) between EE and SC mice indicate that EE determines also a decrease in the spread of the values quantifying the interaction level. This situation implies that a low correlation level represents the preferential status of the M2-V1 connection -resulting in a concentration of the values of the interaction indices in a narrow range- in enriched mice. Conversely, in SC animals the higher correlation level is accompanied by a broader range for the different coupling indices.

One limitation of this study is the relatively low sampling rate, that did not allow us to address the effects of EE in faster (i.e., gamma) frequency bands. However, a decrease of the synchronization level between V1 and M2 cortices in EE animals cannot be accounted for by a shift of the LFP signal out of the recording bandwidth; on the contrary, we found that EE consistently increased the power of the low-frequency delta band (Fig. 4). Another potential concern is that the differences in the LFP analysis are related to a different behavior of EE and standard mice in the recording cage. However, our quantitative analysis showed that the exploratory activity during LFP recordings of enriched mice does not significantly differ from standard animals (Fig. 4). Thus, these observations strongly suggest that the differences in LFP synchronization reflect long-lasting, EE-induced changes in cortical circuitry, rather than an acute response to the recording cage.

A decrease of cortical synchronization in EE animals is consistent with recent results reported by Poulet and Petersen [30]. State-dependent membrane potential synchrony was observed between neurons of the barrel cortex of behaving mice. In particular, high correlation values were observed during quiet whisking period and reduced values during active whisking states [30]. Moreover this effect was observed in the low frequency range (<40 Hz, which is analogous to our LFP sampling range). These data imply that the local activity of small groups of neurons shows a high synchronization with the global brain activity (as measured with the electroencephalogram) as long as they are not engaged in their specific physiological role (e.g. receiving somatosensory signals), during which they otherwise display their own functional identity and a lower correlation with the electroen-cephalogram. In our case, the increased motor and sensory stimulation provided by EE could result in a sustained level of specific activity of the neuronal populations serving a given sensory modality or motor function, which would be responsible, in turn, for a lower degree of correlation between the local field potentials. A lower degree of correlation between V1 and M2 can also be explained by the finding that EE causes a net reduction of GABAergic inhibition in V1 [9], [10]. Indeed, under conditions of reduced inhibition, local activities in V1 and M2 could fluctuate with lower correlation level.

In conclusion, we provide for the first time evidence that an enhanced sensorimotor experience shapes the brain by affecting the functional crosstalk between different cortical areas.

## Supporting Information

Table S1 **Cortical areas showing monosynaptic connections with primary visual cortex.** The table shows a qualitative assessment of the monosynaptic connectivity between primary visual cortex and other cortical areas. The symbols “+, ++, +++” indicate increasing density of CTB-positive somata, whereas “o” indicates no stained cells. Abbreviations in parentheses refer to the Atlas by Paxinos and Franklin [20].
(XLS)

## References

[1] Bavelier D, Neville HJ (2002). Cross-modal plasticity: where and how?. Nat Rev Neurosci.

[2] Singer W (1999). Neuronal synchrony: a versatile code for the definition of relations?. Neuron 24: 49–65,.

[3] Piche M, Chabot N, Bronchti G, Miceli D, Lepore F (2007). Auditory responses in the visual cortex of neonatally enucleated rats.. Neuroscience.

[4] Cohen LG, Celnik P, Pascual-Leone A, Corwell B, Falz L (1997). Functional relevance of cross-modal plasticity in blind humans.. Nature.

[5] Rosenzweig MR (1966). Environmental complexity, cerebral change, and behavior.. Am Psychol.

[6] Rosenzweig MR, Bennett EL (1969). Effects of differential environments on brain weights and enzyme activities in gerbils, rats, and mice.. Dev Psychobiol.

[7] van Praag H, Kempermann G, Gage FH (2000). Neural consequences of environmental enrichment.. Nat Rev Neurosci.

[8] Rosenzweig MR, Bennett EL (1996). Psychobiology of plasticity: effects of training and experience on brain and behavior.. Behav Brain Res.

[9] Sale A, Berardi N, Maffei L (2009). Enrich the environment to empower the brain.. Trends Neurosci.

[10] Mainardi M, Landi S, Gianfranceschi L, Baldini S, De Pasquale R (2010). Environmental enrichment potentiates thalamocortical transmission and plasticity in the adult rat visual cortex.. J Neurosci Res.

[11] Cancedda L, Putignano E, Sale A, Viegi A, Berardi N (2004). Acceleration of visual system development by environmental enrichment.. J Neurosci.

[12] Bartoletti A, Medini P, Berardi N, Maffei L (2004). Environmental enrichment prevents effects of dark-rearing in the rat visual cortex.. Nat Neurosci.

[13] Abarbanel HDI (1996). Analysis of observed chaotic data.. Springer-Verlag.

[14] Boccaletti S, Kurths J, Osipov G, Valladares DL, Zhou CS (2002). The synchronization of chaotic systems.. Physics Reports.

[15] Kantz H, Schreiber T (1997). Nonlinear time series analysis: Cambridge University Press.

[16] Kreuz T, Mormann F, Andrzejak RG, Kraskov A, Lehnertz K (2007). Measuring synchronization in coupled model systems: a comparison of different approaches.. Physica D.

[17] Mormann F, Andrzejak RG, Kreuz T, Rieke C, David P (2003). Automated detection of a preseizure state based on a decrease in synchronization in intracranial electroencephalogram recordings from epilepsy patients.. Phys Rev E Stat Nonlin Soft Matter Phys.

[18] Pikovsky A, Rosenblum M, Kurths J (2001). Synchronization..

[19] Antonucci F, Di Garbo A, Novelli E, Manno I, Sartucci F (2008). Botulinum neurotoxin E (BoNT/E) reduces CA1 neuron loss and granule cell dispersion, with no effects on chronic seizures, in a mouse model of temporal lobe epilepsy.. Exp Neurol.

[20] Paxinos G, Franklin K (2008). The mouse brain in stereotaxic coordinates: Elsevier.

[21] Cover T, Thomas J (1991). Elements of information theory: John Wiley and Sons.

[22] Kraskov A, Stogbauer H, Grassberger P (2004). Estimating mutual information.. Phys Rev E Stat Nonlin Soft Matter Phys.

[23] Bedard C, Kroger H, Destexhe A (2006). Does the 1/f frequency scaling of brain signals reflect self-organized critical states?. Phys Rev Lett.

[24] Press WH, Teukolsky SA, Vetterling WT, Flannery BP (1986). Numerical Recipes.. Cambridge University Press.

[25] Faes L, Porta A, Nollo G (2008). Mutual nonlinear prediction as a tool to evaluate coupling strength and directionality in bivariate time series: comparison among different strategies based on k nearest neighbors.. Phys Rev E Stat Nonlin Soft Matter Phys.

[26] Schiff SJ, So P, Chang T, Burke RE, Sauer T (1996). Detecting dynamical interdependence and generalized synchrony through mutual prediction in a neural ensemble.. Phys Rev E Stat Phys Plasmas Fluids Relat Interdiscip Topics.

[27] Nithianantharajah J, Hannan AJ (2006). Enriched environments, experience-dependent plasticity and disorders of the nervous system.. Nat Rev Neurosci.

[28] Will B, Galani R, Kelche C, Rosenzweig MR (2004). Recovery from brain injury in animals: relative efficacy of environmental enrichment, physical exercise or formal training (1990-2002).. Prog Neurobiol.

[29] Guandalini P (1998). The corticocortical projections of the physiologically defined eye field in the rat medial frontal cortex.. Brain Res Bull.

[30] Poulet JF, Petersen CC (2008). Internal brain state regulates membrane potential synchrony in barrel cortex of behaving mice.. Nature.

